# Diallyl trisulfide inhibits migration, invasion and angiogenesis of human colon cancer HT-29 cells and umbilical vein endothelial cells, and suppresses murine xenograft tumour growth

**DOI:** 10.1111/jcmm.12486

**Published:** 2014-11-17

**Authors:** Kuang-Chi Lai, Shu-Chun Hsu, Jai-Sing Yang, Chien-Chih Yu, Jin-Cherng Lein, Jing-Gung Chung

**Affiliations:** a Department of Surgery, China Medical University Beigang HospitalYunlin, Taiwan; b School of Medicine, China Medical UniversityTaichung, Taiwan; c Department of Biological Science and Technology, China Medical UniversityTaichung, Taiwan; d Department of Pharmacology, China Medical UniversityTaichung, Taiwan; e School of Pharmacy, China Medical UniversityTaichung, Taiwan; f Graduate Institute of Pharmaceutical Chemistry, China Medical UniversityTaichung, Taiwan; g Department of Biotechnology, Asia UniversityTaichung, Taiwan

**Keywords:** DATS, migration and invasion, angiogenesis, HUVEC, HT-29 human colon adenocarcinoma cells

## Abstract

Angiogenesis inhibitors are beneficial for the prevention and treatment of angiogenesis-dependent diseases including cancer. We examined the cytotoxic, anti-metastatic, anti-cancer and anti-angiogenic effects of diallyl trisulfide (DATS). In HT29 cells, DATS inhibited migration and invasion through the inhibition of focal adhesion kinase (FAK), extracellular signal-regulated kinase, c-Jun N-terminal kinase and p38 which was associated with inhibition of matrix metalloproteinases-2, -7 and -9 and VEGF. In human umbilical vein endothelial cells (HUVEC), DATS inhibited the migration and angiogenesis through FAK, Src and Ras. DATS also inhibited the secretion of VEGF. The capillary-like tube structure formation and migration by HUVEC was inhibited by DATS. The chicken egg chorioallantoic membrane (CAM) assay indicated that DATS treatment inhibited *ex-vivo* angiogenesis. We investigated the anti-tumour effects of DATS against human colon cancer xenografts in BALB/c^nu/nu^ mice and its anti-angiogenic activity *in vivo*. In this *in-vivo* study, DATS also inhibited the tumour growth, tumour weight and angiogenesis (decreased the levels of haemoglobin) in HT29 cells. In conclusion, the present results suggest that the inhibition of angiogenesis may be an important mechanism in colon cancer chemotherapy by DATS.

## Introduction

Cancer is one of the major causes of death in the human population 1,2. Colorectal cancer is the third ranking cancer for causing death in Taiwan based on the 2010 report of the Department of Health, R.O.C. (Taiwan). Surgery, radiotherapy and chemotherapy are widely used treatments of human colorectal cancer 2. To date, these treatments of human colorectal cancer are not satisfactory and the mortality in patients with advanced colorectal cancer remains high. Cancer cells invading adjacent tissues and stimulating neovascularization play an important role in tumour growth and metastasis 3,4. Tumours can stimulate the formation of new vessels; without such stimulation they cannot grow beyond 1–2 mm and cannot metastasize 5. Therefore, angiogenesis is an important determinant of tumour progression and it is also a potential target of cancer therapy 6,7. Angiogenesis is the growth of new capillaries from pre-existing capillaries and post-capillary. Tumour growth is angiogenesis-dependent and every increment of tumour growth requires an increment of vascular growth 8,9. It is well known that many diseases, including diabetes mellitus and inflammation are driven by persistent deregulated angiogenesis 10,11. Alternatively, tumour endothelial cells may divide up to 50 times more than normal endothelial cells and they will become activated through the release of pro-angiogenic growth factors including VEGF, fibroblast growth factor (FGF), the transforming growth factors (TGF-α and TGF-β), tumour necrosis factor, platelet derived growth factor (PDGF), interleukin-8, and angiopoietins from tumour and stromal cells 12,13. Angiogenesis is necessary for growth of the primary tumour as well as metastatic spread and invasion 10. It was reported that angiogenesis can be divided into two major stages: the upstream activators of VEGF synthesis and downstream signalling pathways 14. The upstream activators of VEGF synthesis include nitric oxide, H_2_O_2_, hypoxia inducible factor, oncogenes, PDGF, insulin-like growth factor 1, epidermal growth factor, bFGF and interleukin-6 which will stimulate the cancer cells to release VEGF 15–17. The VEGF will then bind and activate the VEGF receptor of endothelial cells leading to downstream signalling pathways, involving inhibition of apoptosis, degradation of the extracellular matrix and cytoskeletal changes and stimulation associated with motility 18.

Regular consumption of fruit and vegetables can reduce certain cancers 19,20. Garlic (*Allium sativum*), for example, has been shown to reduce deaths caused by malignant diseases 21. The major components of garlic include diallyl sulphide (DAS), diallyl disulfide (DADS) and diallyl trisulfide (DATS) and these compounds can inhibit neoplastric cell growth 22. We have demonstrated that DATS > DADS > DAS in affecting drug resistance, migration, invasion and caused cell death in human colon cancer colo 205 cells 23. DAS, DADS and DATS differ in their magnitude of effects which is related to the number of sulphur atoms in each compound 24. Although DAS and DADS have been shown to have antiangiogenic activity 25,26, effects of DATS on angiogenesis have not been reported. Therefore, the purpose of this study was to determine effects of DATS on angiogenesis in human colon cancer cells *in vitro* and in human umbilical vein endothelial cells (HUVEC) *ex vivo*.

## Materials and methods

### Chemicals and regents

Diallyl trisulfide was purchased from LKT Laboratories (St. Paul, MN, USA), and it was dissolved in dimethyl sulphoxide (DMSO; Sigma-Aldrich Corp., St. Louis, MO, USA). All antibodies were purchased from Santa Cruz Biotechnology, Inc. (Santa Cruz, CA, USA).

### Cell lines

Human umbilical vein endothelial cells were purchased from the Food Industry Research and Development Institute (Hsinchu, Taiwan), and cultured in 75-cm^2^ plastic tissue flasks then grown at 37°C under a humidified 5% CO_2_ atmosphere in M199 medium (Invitrogen Crop., Carlsbad, CA, USA) supplemented with 20% foetal bovine serum (FBS; Invitrogen Crop.) and 15 μg/ml endothelial cell growth supplements. Confirmation of endothelial cells was performed through the detection of CD31 (PECAM-1) by flow cytometry. The human colon adenocarcinoma cell line (HT-29) was purchased from the Food Industry Research and Development Institute. HT-29 cells were placed separately in 75 cm^3^ tissue culture flasks and grown at 37°C under a humidified 5% CO_2_ atmosphere in RPMI 1640 medium (Invitrogen Crop.) supplemented with 10% FBS, 100 Units/ml penicillin and 100 μg/ml streptomycin (Invitrogen Crop.).

### Determination of cell viability

Approximately, 2 × 10^5^ cells/well of HT-29 or HUVEC were maintained in 12-well plates and then were incubated without (1% DMSO vehicle control) or with 1.5625, 3.125, 6.25, 12.5, 25 or 50 μM of DATS for 24 hrs. Cells from each treatment were harvested for viability determination, and harvested cells were stained with PI (5 μg/ml) and then analysed with a flow cytometer equipped with an argon ion laser at 488 nm wavelength (Becton-Dickinson, San Jose, CA, USA) as previously described 27.

### Cell migration assay

The 24-well Transwell inserts with 8 μm porosity polycarbonate filters (Millipore Corp., Billerica, MA, USA) were coated with 30 μg type I collagen (Millipore) for 1 hr at room temperature to form a genuine reconstituted basement membrane. HT-29 cells (10^4^ cells/0.4 ml medium) were placed in the upper compartment and exposed to 1.56 and 3.12 μM of DATS. Cells then were incubated at 37°C for 24 and 48 hrs. At the end of the incubation, cells were fixed with 4% paraformaldehyde and stained with 2% crystal violet. The cells on the upper surface of the filter were removed by wiping with a cotton swab, and the cells that penetrated through the collagen to the lower surface of the filter were counted under a light microscope at 200× magnification 28.

### Cell invasion assay

The 24-well Transwell inserts with 8 μm porosity polycarbonate filters (Millipore) were coated with 30 μg Matrigel (BD Biosciences, San Jose, CA, USA) at room temperature for 1 hr to form a basement membrane. HT-29 cells (10^4^ cells/0.4 ml medium) were placed in the upper compartment and exposed to 1.56 and 3.12 μM of DATS. Cells then were incubated at 37°C for 24 and 48 hrs. The cells were fixed with 4% paraformaldehyde and stained with 2% crystal violet. Cells on the upper surface of the filter were removed by wiping with a cotton swab, and the cells that penetrated through the matrigel to the lower surface of the filter were counted under a light microscope at 200× magnification 29.

### Determinations of VEGF secretion by ELISA

HT-29 cells (approximately, 5 × 10^5^ cells/well) were placed in 12-well plates and treated with 0, 1.56, 3.12 and 6.25 μM of DATS for 24 hrs-incubation, after which the supernatant was collected by centrifugation. All samples were assayed for VEGF concentrations by ELISA assay kits (R&D Systems, Minneapolis, MN, USA) that were used according to the manufacturer's recommendations. Absorbance was monitored at 450 nm with a reference wavelength of 570 nm with an ELISA reader 30.

### Real-time PCR of *MMP-2, -7 -9 and VEGF* genes levels

Total RNA was extracted from HT-29 cells after treatment with 0, or 12.5 μM of DATS for 24 or 48 hrs, using the Qiagen RNeasy Mini Kit (Qiagen, Valencia, CA, USA) as previously described 31. Briefly, the RNA samples from each treatment were reverse-transcribed for 30 min. at 4°C with High Capacity cDNA Reverse Transcription Kit according to the standard protocol of the supplier (Applied Biosystems, Foster City, CA, USA). Quantitative PCR was performed with the following conditions: 2 min. at 50°C, 10 min. at 95°C, and 40 cycles of 15 sec. at 95°C, 1 min. at 60°C using 1 μl of the cDNA reverse-transcribed as described above, 2× SYBR Green PCR Master Mix (Applied Biosystems) and 200 nM of forward (F) and reverse (R) primers.

MMP-2-F: CCCCAGACAGGTGATCTTGAC, MMP-2-R: GCTTGCGAGGGAAGAAGTTG; MMP-7-F: GGATGGTAGCAGTCTAGGGATTAACT, MMP-7-R: AGGTTGGATACATCACTGCATTAGG; MMP-9-F: CGCTGGGCTTAGATCATTCC, MMP-9-R: AGGTTGGATACATCACTGCATTAGG; VEGF-F: CTTGCCTTGCTGCTCTACCT, VEGF-R: TGATGTTGGACTCCTCAGTGG; GAPDH-F: ACACCCACTCCTCCACCTTT, GAPDH-R: TAGCCAAATTCGTTGTCATACC.

Each assay was run on an Applied Biosystems 7300 Real-Time PCR system in triplicate and expression fold-changes were derived using the comparative C_T_ method 28,31.

### Western blotting analysis

HT-29 cells were placed in 6-well plates treated with or without 12.5 μM of DATS for 0, 6, 12, 24 and 48 hrs. HUVEC were seeded in 6-well plates and exposed to 12.5, 25 and 50 μM of DATS for 24 hrs. Cells were harvested and centrifuged, and the isolated cells were washed twice with PBS. The cells were lysed in 200 μl of the PRO-PREP protein extraction solution (iNtRON Biotechnology, Seongnam, Gyeonggi-Do, Korea) for 2 hrs. The samples were then centrifuged at 12,000 × g for 10 min. at 4°C. The supernatants were collected and the protein levels were determined by the Bio-Rad detergent-compatible protein assay kit (Bio-Rad, Hercules, CA, USA) with bovin serum albumin (BSA) as a standard 31,32. Proteins (40 μg/lane) from each sample were individually separated on 12% SDS-polyacrylamide gels and blotted onto polyvinyliene difluoride (PVDF, Immobilon-P Transfer Membrane, Millipore) membranes. The membranes were individually incubated with 5% BSA and primary antibodies (anti-iNOS, Cox II, uPA, Ras, RhoA, FAS, SOS, PI3K, MKK7, MEKK3, extracellular signal-regulated kinase 1/2 (ERK1/2), c-Jun N-terminal kinase 1/2 (JNK1/2), p38, ROCK-1, Src, focal adhesion kinase (FAK), protein kinase C α (PKCα), p-ERK1/2, ERK, H-Ras, K-Ras, N-Ras) overnight at 4°C. Blots were then washed three times in PBS with 0.04% Tween-20 (PBST) for 5 min. before being incubated with horseradish peroxidase (HRP)-conjugated secondary antibody at 1:1000 dilutions in PBST containing 5% milk for 2 hrs at room temperature. The membranes were washed with PBST and were visualized using ECL kit (Immobilon Western HRP substrate, Millipore) and were quantified by densitometry using Image J image analysis 31,32.

### Wound healing assay

Human umbilical vein endothelial cells migration was measured by using a wound-healing assay. HUVEC (1 × 10^5^ cells/well) were placed for 24 hrs in six-well plates and at confluence a wound was made by using a pipette tip and cell debris was removed by washing with serum-free medium. Cells on the plates were then photographed under phase-contrast microscope (time = 0) and then incubated in media with or without DATS (0 and 25 μM) at 37°C and 5% CO_2_ and allowed to migrate into the wound area for up to 24 hrs. Cells were then gently washed with PBS, and the wound area was photographed under phase-contrast microscope 33.

### Tube formation assay

Matrigel (30 μl) was pipetted into a 24-well flat bottomed plate and kept for 30 min. at 37°C. HUVEC (2 × 10^5^ cells) were seeded into the layer of polymerized Matrigel with or without 12.5 and 25 μM of DATS and VEGF in a chamber slide (Nalge Nunc International, Naperville, IL, USA). Matrigel cultures were incubated for 24 hrs at 37°C in 5% CO_2_ atmosphere. Following incubation, the tube formation was visualized, evaluated and photographed using a phase-contrast microscope (200× magnification) 34.

### Chick chorioallantoic membrane assay for angiogenesis

Effects of DATS on *ex vivo* angiogenesis was measured by the chorioallantoic membrane (CAM) assay. Fertilized White Leghorn chicken eggs were incubated at 37°C under conditions of constant humidity. On embryonic day 6, the developing CAM was separated from the shell by opening a small circular window at the broad end of the egg above the air sac. The opening was sealed with Parafilm, and the eggs were incubated for two more days. DATS (6.25 μM) was prepared in PBS supplemented with 30 ng/ml of VEGF. On day 8, 20 μl was loaded onto 2-mm^3^ gelatin sponges (Gelfoam; Pharmacia and Upjohn Co., Donmills, Canada) that were placed on the surface of the developing CAM. Sponges containing vehicle alone (20 μl of PBS) were used as negative controls, whereas sponges containing 20 μl of 30 ng/ml of VEGF in PBS were used as positive controls. Eggs were resealed and returned to the incubator. On day 10, images of CAM were captured digitally using an Olympus SZX9 (Olympus; Tokyo, Japan) stereomicroscope equipped with a Spot RT digital imaging system (Diagnostic instruments Inc., Sterling Heights, MI, USA) 35.

### BALB/c^nu/nu^ mouse HT-29 xenograft model *in vivo*

Male nude mice (BALB/c^nu/nu^) at 6–8 weeks of age were obtained from the National Laboratory Animal Center (Taipei, Taiwan). All animals were maintained in standard vinyl cages with air filter tops in a filtered laminar air flow room at 25°C on a 12-hr light/dark cycle. Water and food were autoclaved and provided for all animals. HT-29 cells (5 × 10^6^) in RPMI-1640 medium were subcutaneously injected into the flanks of mice. Tumour-bearing mice were then randomly divided into treatment groups (ten mice per group) and treatment initiated when the xenografted solid tumours reached a volume of about 100 mm^3^. Each mouse received either 100 μl of control vehicle (olive oil), or DATS (10 and 50 mg/kg) by oral administration daily beginning at day 4 and ending on day 32. All experiments were conducted according to institutional guidelines and approved by the Animal Care and Use Committee of China Medical University. After xenograft transplantation, mice exhibiting tumours were monitored and tumour size was measured once every 4 days using callipers. The tumour volume in each animal was estimated according to the formula: tumour volume (mm^3^) = *L* × *W*^2^/2 (where *L* is the length and *W* is the width) with the final measurement taken 4 weeks after tumour cell inoculation. Body weights of all mice were measured once every 4 days. At the end of the experiment (4 weeks after cell inoculation), animals were anaesthetized by CO_2_ and killed. Tumours from each animal were removed, measured and weighted 36.

### Angiogenesis assay *in vivo*

Tumours from each animal were removed, and haemoglobin was measured as an indication of blood vessel formation using the Drabkin method (Drabkin reagent kit 525; Sigma-Aldrich) 34. The concentration of haemoglobin was calculated from a known amount of haemoglobin assayed in parallel.

### Statistical analysis

Student's *t*-test was used to analyse differences between treatment and control groups. A statistical significance was shown as follows: *P < 0.05, **P < 0.01 and ***P < 0.001.

## Results

### DATS inhibited the cell migration and invasion of HT-29 cells

**Fig 1:**
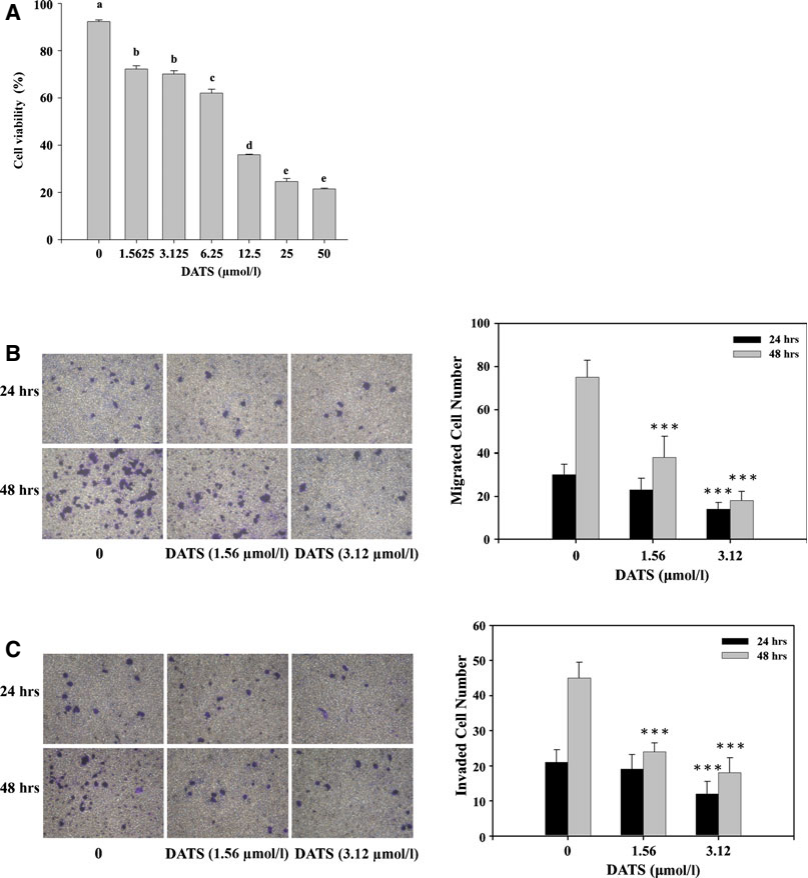
DATS affected the percentage of viable cells and migration and invasion of HT-29 cells *in vitro*. Cells were placed in RPMI1640 medium + 10% FBS with different doses of DATS for 24 hrs to measure the percentages of viable cells (A). The cells were collected and analysed for viability by flow cytometry as described in Materials and methods. Cell migration was examined using Transwell inserts with polycarbonate filters (pore size, 8 μm; B). Cell invasion was examined in Transwell inserts with polycarbonate filters (pore size, 8 μm) pre-coated with matrigel (C). Figure 1A used anova analysis for Statistical analysis. Different letters (a-e) represent statistically significant difference among treatments (*P* < 0.05). Migration and invasion ability of HT-29 cells were quantified by counting the number of cells that invaded the underside of the porous polycarbonate membrane under microscopy and represent the average of three experiments. Figures 1B and C were used Student's *t*-test for control and treated group. A statistical significance was shown as follows: *P < 0.05, **P < 0.01 and ***P < 0.001.

Results from the flow cytometric assay are shown in Figure1A, which indicated that DATS-induced cytotoxic effects in HT-29 cells in a concentration-dependent manner. As shown in Figure1, there were fewer viable cells (*P* < 0.001) as DATS concentration increased from 1.5625 to 50 μM when compared with the control group. Next, we determined the effects of DATS treatment on migration and invasion of HT-29 cells by using a Boyden chamber assay. In control sample, a large fraction of HT-29 cells migration and invasion to the bottom face of the membrane was decreased markedly in the presence of DATS. DATS significantly inhibited the HT-29 cell migration and invasion in a time- and concentration-dependent manner (Fig.1B and C).

### DATS inhibited *MMP-2, MMP-7, MMP-9* and *VEGF* secretion in HT-29 cells

**Fig 2:**
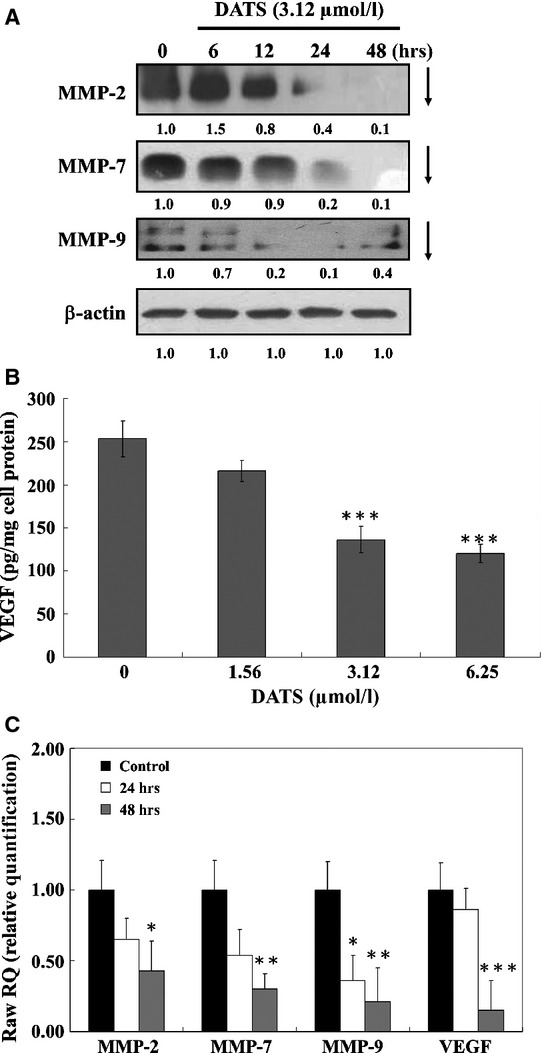
DATS affected the level of proteins of associated migration and invasion MMP-2, -7 and -9 mRNA expressions in HT-29 cells. Cells were treated with 0, 3.12 μM of DATS for 24 and 48 hrs. The total proteins were collected and the proteins levels (MMP-2,-7 and -9) were examined by Western blot (A) as described in Materials and methods. Cells were treated with 0, 1.56, 3.12 and 6.25 μM of VEGF by ELISA assay (B) as described in Materials and methods. Cells were treated with 3.12 μM DATS then were incubated for 24 and 48 hrs. The total RNA was extracted and RNA samples were reverse-transcribed cDNA then for real-time PCR assay (C) as described in ‘Materials and methods’. The ratios of MMP-2,-7 and VEGF mRNA/GAPDH are presented. Data represent mean ± SD of three experiments. *P < 0.05, **P < 0.01, ***P < 0.001 were considered significantly.

We investigated the mechanisms of this cell invasive phenotype by looking at the involvement of matrix metalloproteinases (MMP)-2, -7, -9 and VEGF by Western blot and ELISA. As shown in Figure2A, DATS inhibited protein levels of MMP-2, MMP-7 and -9 in a time-dependent manner. The VEGF secretion was also reduced in HT-29 cells after treatment with DATS (Fig.2B). As shown in Figure2B, DATS-mediated secretion of VEGF was evident at 3.12 μM DATS compared to the control, and the secretion of VEGF into the medium was inhibited by 56% and 48% at a 24 hrs treatment with 3.12 and 6.25 μM DATS, respectively (Fig.2B). Real-time PCR also showed that DATS inhibited mRNA expression of MMP-2, MMP-7, MMP-9 and VEGF in HT-29 cells after a 48 hrs treatment (Fig.2C).

### DATS affected the upstream signal of the metastasis associated proteins in HT-29 cells

**Fig 3:**
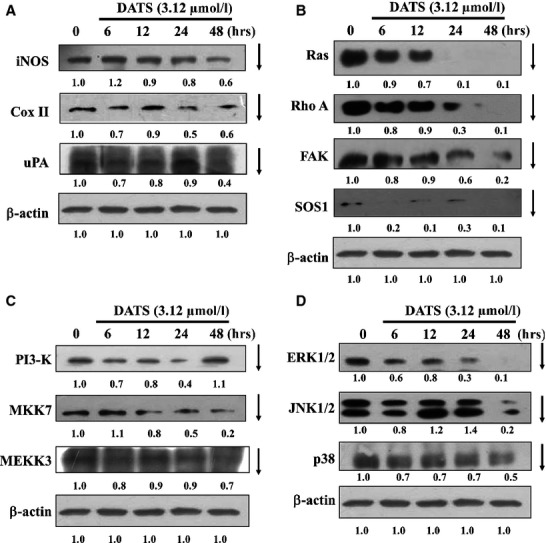
DATS affected on the levels of up-signals proteins associated with migration and invasion in HT-29 cells. Cells were treated with 3.12 μM of DATS for 0, 6, 12, 24 and 48 hrs. The total proteins were collected and the proteins levels iNOS, COX II, uPA (A); Ras, Rho A, FAK and SOS (B); PI3-K, MKK7 and MEKK3 (C); ERK1/2, JNK1/2 and p38 (D) were examined by Western blot as described in Materials and methods.

**Fig 4:**
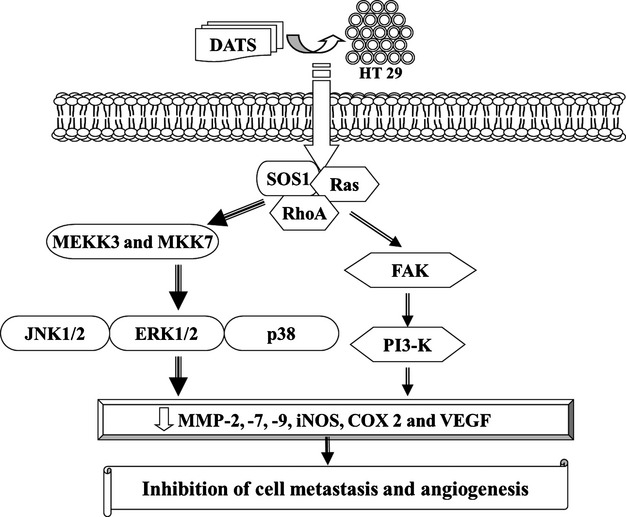
Proposed signal pathways of DATS inhibited the migration and invasion of HT-29 cells *in vitro*.

To examine the possible signalling pathways for DATS inhibiting metastasis of HT-29 cells, the cells were treated with 12.5 μM of DATS for different time periods to observe changes of associated protein levels by Western blot. Results are shown in Figure3A–D. DATS-reduced proteins levels of iNOS, Cox II, uPA (Fig.3A), Ras, RhoA, FAK, SOS (Fig.3B), PI3-K, MKK7, MEKK3 (Fig.3C), ERK, JNK and p38 (Fig.3D). These results suggest that DATS down-regulated upstream signalling proteins followed by inhibition MMP-2, -7, -9 and VEGF secretion which was associated with inhibition of migration and invasion. The possible signal pathways for DATS inhibited the migration and invasion of HT-29 cells are shown in Figure4.

### DATS-inhibited HUVEC migration

**Fig 5:**
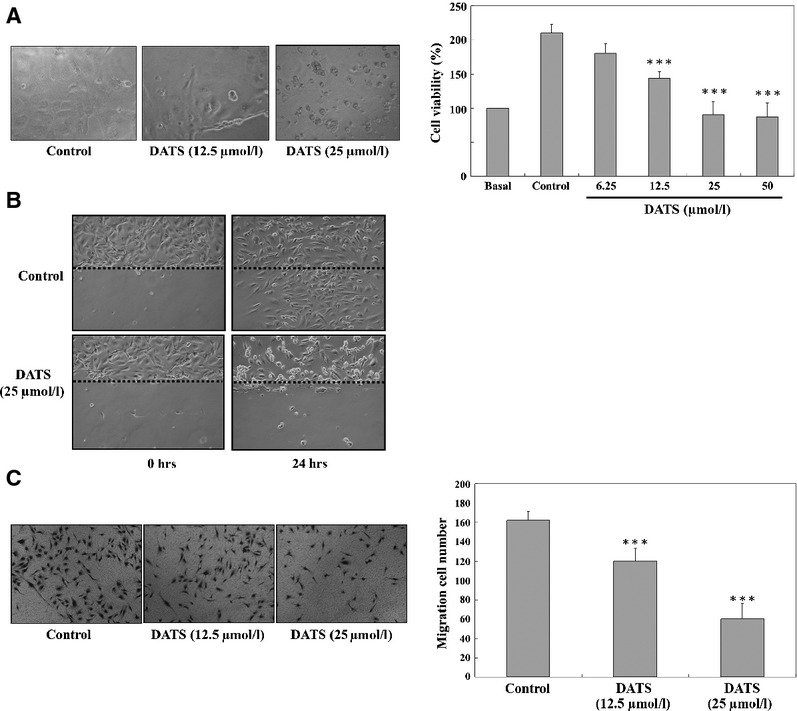
DATS decreased the percentage of viable cells, and inhibited the cells migration of HUVEC. Cells were treated with the supernatant of DATS-treated HT-29 cells before HUVEC were examined for the morphological changes and the percent of viable cells (A) and the migration *in vitro* by wound healing examination (B) and Transwell migration assay (C) as described in Materials and methods. Data represent mean ± SD of three experiments. ***P < 0.001 was considered significant.

We determined effects of DATS-treated HT-29 supernatant on inhibition of HUVEC growth. Results are shown in Figure5A–C. Figure5A indicated that DATS significantly inhibited HUVEC viability in a concentration-dependent manner. To examine whether DATS affected the migratory behaviour of HUVEC, we performed a wound healing assay and a transwell migration assay. Results of the wound healing assay showed that DATS significantly inhibited HUVEC migration (Fig.5B) and as seen in Figure5C, DATS inhibited cell migration in a dose-dependent manner.

### Effects of DATS on VEGF-induced tube formation in HUVEC and an *in vivo* CAM assay

**Fig 6:**
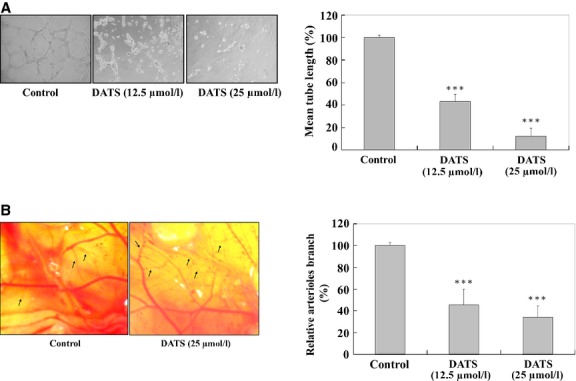
DATS affected on VEGF-induced tube formation of HUVEC and an *in vivo*CAM assay. HUVEC (2 × 10^5^ cells) were incubated with or without 12.5 and 25 μM of DATS and then seeded in a 96-well culture plate pre-coated with Matrigel (BD Biosciences) and then were incubated for 24 hrs at 37°C in 5% CO_2_ atmosphere. After incubation, the cells morphology were evaluated by using a phase-contrast microscope and were photographed (200 × ; A). The quantitative data were determined using Image analysis software (B). On embryonic day 6 of fertilized White Leghorn chicken eggs, the developing CAM was separated from the shell by opening a small circular window at the broad end of the egg above the air sac. The eggs were incubated for two more days. Twenty-five μM DATS was prepared in PBS supplemented with 30 ng/ml of VEGF. On day 8, 20 μl was loaded onto 2-mm^3^ gelatin sponges as described in Materials and methods. Eggs were resealed and returned to the incubator. On day 10, images of CAM were captured digitally using an Olympus SZX9 stereomicroscope equipped with a Spot RT digital imaging system (C). The quantitative data indicated that the concentration of DATS was significantly different compared with control (D). ***P < 0.001 was considered significant.

Formation of new blood vessels requires endothelial cells to invade the extracellular matrix, raising the possibility that DATS also inhibits angiogenesis. Effects of DATS on angiogenesis *ex vivo* was tested using an *in vitro* tube formation assay and an *ex vivo* CAM assay. DATS (12.5 and 25 μM) of caused a concentration-dependent blockage of the capillary tubes (Fig.6A and B). Microscopic examination of the CAM revealed highly vascularized structure among tabulate of the control group (Fig.6B). In the CAM assay, angiogenesis in response to VEGF (30 ng/ml) was reduced in embryos by treatment with 25 μM of DATS (Fig.6B). Results of the CAM assay demonstrated that DATS has the ability to block VEGF-induced *in vitro* and *in vivo* angiogenesis (Fig.6A and B).

### DATS affected angiogenesis associated proteins in HUVEC

**Fig 7:**
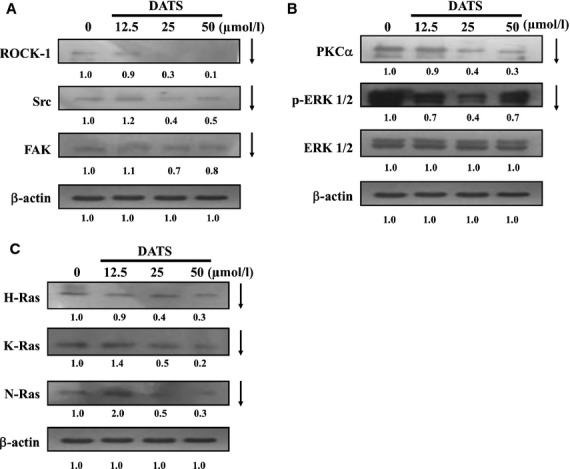
DATS affected the upstream signal of the angiogenesis associated proteins in HUVEC. Cells were treated with 0, 12.5, 25, 50 μM of DATS for 24 hrs. The total proteins were collected and the proteins levels (A: ROCK-1, Src and FAK; B: PKCα, p-ERK1/2 and ERK; C: H-Ras, K-Ras and N-Ras) were examined by SDS-PAGE and Western blot as described in Materials and methods.

**Fig 8:**
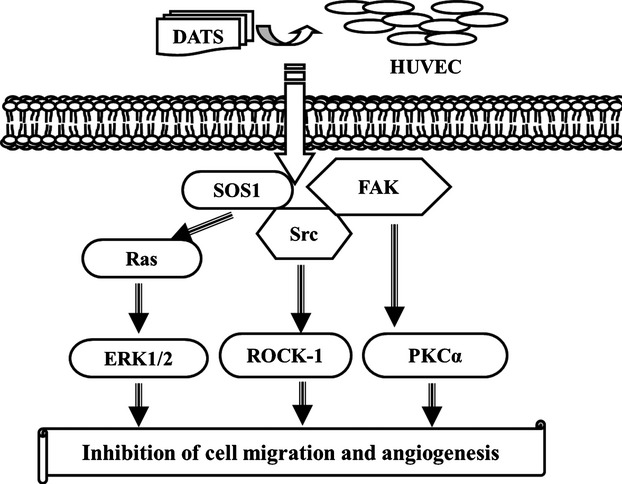
Proposed signal pathways of DATS inhibited the angiogenesis associated proteins in HUVEC.

To examine the possible signalling pathways of DATS inhibition of angiogenesis in HUVEC *in vitro*, the cells were treated with DATS (0, 12.5, 25 and 50 μM) of for 24 hrs and in and protein levels determined by Western blots. Results are shown in Figure7A–C. DATS reduced protein levels of ROCK-1, Src, FAK (Fig.7A), PKCα, p-ERK (Fig.7B), H-Ras, K-Ras and N-Ras (Fig.7C). These results suggest that DATS down-regulated upstream signalling proteins associated with inhibition of angiogenesis. The possible signalling pathways for DATS inhibiting the angiogenesis of HUVEC are summarized in Figure8.

### BALB/c^nu/nu^ mouse HT-29 xenograft model *in vivo*

**Fig 9:**
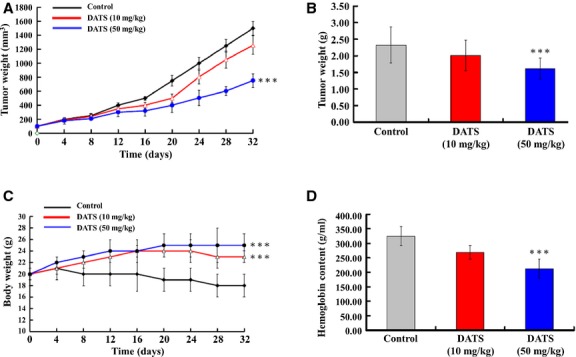
DATS affected HT29 tumour and angiogenesis in BALB/cnu/nu mouse HT29 xenograft model *in vivo*. HT-29 cells (5 × 10^6^) in RPMI-1640 medium were subcutaneously injected into the flanks of BALB/c^nu/nu^ mice. Tumour-bearing mice were then randomly divided into treatment groups (ten mice per group) and treatment initiated when the xenografted solid tumours reached a volume of about 100 mm^3^. Each mouse was orally receiving, every day, either 100 μl of control vehicle (olive oil), or DATS (10 and 50 mg/kg). The tumour volume in each animal was estimated according to the formula: tumour volume (mm^3^) = L × W^2^/2 (where L is the length and W is the width) with the final measurement taken 4 weeks after tumour cell inoculation (A). At the same time, the body weight of each animal was measured once every 4 days (C). At the end of the experiment (4 weeks after cell inoculation), animals were anaesthetized by CO_2_ and killed. Tumours from each animal were removed, measured and weighted individually (B). Tumours from each animal were removed, haemoglobin was measured as an indication of blood vessel formation, using the Drabkin method (D). The concentration of haemoglobin was calculated from a known amount of haemoglobin assayed in parallel. ***P < 0.001 was considered significant.

On the basis of our *in-vitro* studies, we further examined the *in-vivo* anti-colon cancer activities of DATS in a BALB/c^nu/nu^ mouse HT-29 xenograft model. As shown in Figure9A, DATS (10 and 50 mg/kg) reduced tumour volume compared to control groups. Representative tumour weight in the HT-29 xenograft mice treated with or without DATS are shown in Figure9B; DATS significantly decreased the tumour weight compared to control groups. Body weights of the xenograft mice were not significantly changed after DATS (10 and 50 mg/kg) treatment when compared with control groups (Fig.9C). However, the control group of mice was only injected by HT-29 cells, and the body weight was decreased from day 8th to 32nd. Both DATS doses (10 and 50 mg/kg) significantly prevented the loss of body weight as compared with control group. The concentration of haemoglobin of tumour sections significantly decreased in DATS (50 mg/kg) treated-HT-29 xenograft mice as compared with the control group. Our results suggest that DATS causes anti-tumour and anti-angiogenesis activities in a HT-29 xenograft animal model *in vivo*.

## Discussion

Diallyl trisulfide is one of the major organosulphur compounds of garlic, and it has been recognized to have antioxidant, antiproliferative and anticarcinogenic properties 37. Studies *in-vivo* found that oral administration of DATS inhibited the growth, metastasis and angiogenesis of orthotopically implanted human prostate PC-3 tumours 38. Although anti-cancer effects of DATS have been demonstrated, there is no information about the effects of DATS on angiogenesis in human colon cancer cells. In this study, our results demonstrated that DATS decreased cell numbers of human colon cancer HT-29 cells and HUVEC in a dose- and time-dependent manner. DATS inhibited the migration and invasion of HT-29 cells and HUVEC. This is the first report to show that DATS reduces the growth of HT-29 cell tumour xenografts with a decrease in blood vessel density (Fig.9), suggesting that one mechanism whereby DATS decreases tumour cell proliferation is *via* reducing angiogenesis. Results also showed that DATS inhibited tubule formation and reduced the number of blood vessels in a CAM experiments (Fig.6). Furthermore, we found that DATS markedly reduced VEGF in both human colon HT29 cells and HUVEC, suggesting that VEGF is involved in the anti-angiogenic responses of DATS. We propose that the inhibition of angiogenesis maybe one of the major mechanisms of DATS induced cancer chemoprevention.

It is well known that metastases formation is a major factor in disease progression and accounts for the majority of cancer deaths 39,40. Recently, the invasion of tumour cells into Matrigel has been widely used for characterizing the involvement of extracellular matrix receptors and matrix degrading enzymes, in tumour progression. Our *in-vitro* Matrigel invasion assay demonstrated marked impairment of the invasive capability of HT29 cells after treatment with DATS. It is well known that cell migration and invasion involve many factors and to examine which of these associated factors are affected by DATS, we looked at specific protein levels. DATS reduced proteins levels of iNOS, Cox II, uPA (Fig.3A), Ras, RhoA, FAK, SOS (Fig.3B), PI3-K, MKK7, MEKK3 (Fig.3C), ERK, JNK and p38 (Fig.3D) in HT29 cells. These results suggest that DATS down-regulated upstream signalling proteins followed by reducing protein secretion of MMP-2, -7, -9 (Fig.2A) which was associated with the inhibition of migration and invasion. Furthermore, real time PCR assays also showed that DATS inhibited mRNA expression of MMP-2, -7, -9 and VEGF (Fig.2C).

Exposure of HUVEC to DATS caused suppression of VEGF secretion to the medium (Fig.2B). The precise molecular mechanism(s) for the DATS-mediated decrease in VEGF is not known. Because VEGF mRNA of HT29 cells after treatment with DATS was also reduced, DATS may activate signalling pathways to inhibit VEGF transcription. VEGF expression has been reported to be regulated at transcription by RNA stability, at translation by mRNA capping proteins, and at post-translation by glycosylation 41. VEGF was reported to be a proangiogenic growth factor most closely associated with aggressive human cancer cells 42. Therefore, the precise molecular mechanism(s) for the DATS-mediated decrease in VEGF secretion is currently under investigation.

Our results also showed that DATS reduced the protein levels of COX-II (Fig.3A) and MMP-2 and-9 (Fig.2A) in HT-29 cells. It was reported that the overexpression of COX-II is associated with the initiation of angiogenesis and COX-2 inhibitors can block angiogenesis 30. COX-2 inhibitors suppress the expression and secretion of MMP-2 and-9, suggesting that COX-2 also promotes tumour angiogenesis and tumour invasion. Furthermore, it was also reported that the downstream products of COX-2 stimulate VEGF to promote angiogenesis 30. Taken together, our studies suggest that the decrease in angiogenesis by DATS may be due to the reduction in COX-2, VEGF which leads to the inhibition of MMP-2, -7 and -9 and inhibition of migration and invasion (Fig.4).

The ability of colon cancer cells to induce angiogenesis plays an important role in tumour invasion and metastasis Development of effective therapeutic strategies for blockage of angiogenesis is required. Angiogenesis is one of the physiological processes involving the growth of new blood vessels from pre-existing vessels and it is required for tumour growth and metastasis 43. Angiogenesis is regulated by multiple factors 44 such as VEGF, FGF, and hepatocyte growth factor. It was reported that VEGF plays a key role in angiogenesis and tumour cell metastasis 45,46. VEGF is the primary proangiogenic factor released from cancer cell leading to endothelial proliferation, survival, and tube formation 47,48. Inhibition of angiogenesis is cancer treatment 49. In addition to VEGF, we also found out that DATS decreased the protein levels of PKC in HT-29 cells. VEGF mainly binds to its receptor, KDR/Flk-1, and activates MAP kinase *via* PKC. PKC inhibitors have been reported to inhibit angiogenesis 50. We found that DATS inhibited the growth of colon cancer *in vivo* in the subcutaneous xenograft model at 50 mg/kg: at this dose, DATS had a significant reducing effect on tumour growth.

Our results also showed that DATS inhibited the secretion of VEGF in HT29 cells. These observations support the hypothesis that DATS may inhibit HUVEC angiogenesis through the suppression of VEGF-mediated signalling pathways. We also found that DATS alone significantly inhibited the protein levels and gene expression mRNA levels of MMP-2, MMP-7, MMP-9 and VEGF in HT-29 cells. Based on the results of this study, we propose the signalling pathways of DATS-inhibited metastasis in HT-29 cells and DATS-inhibited angiogenesis in HUVEC which are shown in Figure6B. Taken together, our results suggest that DATS is a potent angiogenesis inhibitor with the potential to become a useful agent in the treatment of human colon cancer and other angiogenesis-dependent diseases.
